# The predictive value of *γ*-glutamyl transferase to serum albumin ratio in hepatocellular carcinoma patients after liver transplantation

**DOI:** 10.3389/fmed.2024.1380750

**Published:** 2024-05-10

**Authors:** Xing-Yu Luo, Kai-Wun Chang, Nan Ye, Chen-Hao Gao, Qing-Bo Zhu, Jian-Peng Liu, Xing Zhou, Shu-Sen Zheng, Zhe Yang

**Affiliations:** ^1^Department of Hepatobiliary and Pancreatic Surgery, Shulan (Hangzhou) Hospital Affiliated to Zhejiang Shuren University Shulan International Medical College, Hangzhou, China; ^2^Key Laboratory of Artificial Organs and Computational Medicine in Zhejiang Province, Shulan International Medical College, Zhejiang Shuren University, Hangzhou, China; ^3^Graduate School, Zhejiang Chinese Medical University, Hangzhou, China; ^4^Department of Hepatobiliary and Pancreatic Surgery, First Affiliated Hospital, School of Medicine, Zhejiang University, Hangzhou, China; ^5^MSK Laboratory, Department of Surgery and Cancer, Faculty of Medicine, Imperial College London, London, United Kingdom

**Keywords:** liver transplantation, hepatocellular carcinoma, gamma-glutamyl transferase to serum albumin ratio, overall survival, prognosis

## Abstract

**Background:**

Elevated preoperative γ-glutamyl transferase (GGT) levels or reduced serum albumin levels have been established as negative prognostic factors for patients with hepatocellular carcinoma (HCC) and various other tumors. Nonetheless, the prognostic significance of the GGT to serum albumin ratio (GAR) in liver transplantation (LT) therapy for HCC is still not well-defined.

**Methods:**

A retrospective analysis was conducted on the clinical data of 141 HCC patients who underwent LT at Shulan (Hangzhou) Hospital from June 2017 to November 2020. Using the receiver operating characteristic (ROC) curve, the optimal GAR cutoff value to predict outcomes following LT was assessed. Univariate and multivariate Cox proportional hazards regression analyses were used to identify independent risk factors associated with both overall survival (OS) and recurrence-free survival (RFS).

**Results:**

A GAR value of 2.04 was identified as the optimal cutoff for predicting both OS and RFS, with a sensitivity of 63.2% and a specificity of 74.8%. Among these patients, 80 (56.7%) and 90 (63.8%) met the Milan and the University of California San Francisco (UCSF) criteria, respectively. Univariate Cox regression analysis showed that microvascular invasion (MVI), maximum tumor size (>5 cm), total tumor size (>8 cm), liver cirrhosis, TNM stage (III), and GAR (≥2.04) were significantly associated with both postoperative OS and RFS in patients with HCC (all *p* < 0.05). Multivariate Cox regression analysis indicated that GAR (≥2.04) was independently linked with RFS and OS.

**Conclusion:**

Pre-transplant GAR ≥2.04 is an independent correlate of prognosis and survival outcomes after LT for HCC and can be used as a prognostic indicator for both mortality and tumor recurrence following LT.

## Introduction

Hepatocellular carcinoma (HCC) is a malignancy that significantly affects human health, ranking as one of the most prevalent malignancies globally and the third highest cause of cancer-related deaths (1, 2). Chronic liver disease and cirrhosis cause approximately 2 million deaths worldwide each year. Although there are various strategies and methods currently available for treating HCC and end-stage liver disease, liver transplantation (LT) remains one of the most effective treatments. Indications include irreversible liver damage (i.e., cirrhosis) caused by chronic viral infections, excessive alcohol consumption, and liver cancer or acute liver failure (1, 3). In 1996, the Milan criteria were introduced as guidelines for LT in HCC patients, although they were later considered too strict. Subsequently, a research team from the University of California, San Francisco (UCSF), and Hangzhou, China, proposed more comprehensive standards, including the Hangzhou standard, which incorporated innovative elements such as preoperative serum alpha-fetoprotein (AFP) levels and tumor histological differentiation (4–7). However, it is now understood that for HCC patients undergoing LT, their prognosis is influenced by multiple factors, including graft function, rejection, recurrence, and complications. Statistics indicate a 5-year survival rate of 75–80% following surgery, with a relatively low risk of recurrence at approximately 15% (1). Therefore, it is crucial to identify reliable biomarkers or test indicators to assess the prognosis of patients with HCC.

In recent years, inflammation scores, such as the neutrophil-to-lymphocyte ratio (NLR) (8) and platelet-to-lymphocyte ratio (PLR) (9), have demonstrated the ability to reflect the body’s immunological function and inflammatory status. These indicators not only provide insights into the likelihood of recurrence and early mortality after LT for HCC but are also directly associated with patient survival post-transplantation. Additionally, studies based on preoperative γ-glutamyl transferase (GGT) to serum albumin ratio (GAR) have shown a strong correlation with the prognosis after partial resection of HCC and radical surgery for pancreatic ductal adenocarcinoma (10–12). However, there is limited research on the prognostic significance of GAR in HCC patients undergoing LT, which led to the current study to evaluate its importance.

## Method

### Patients

**Figure 1:**
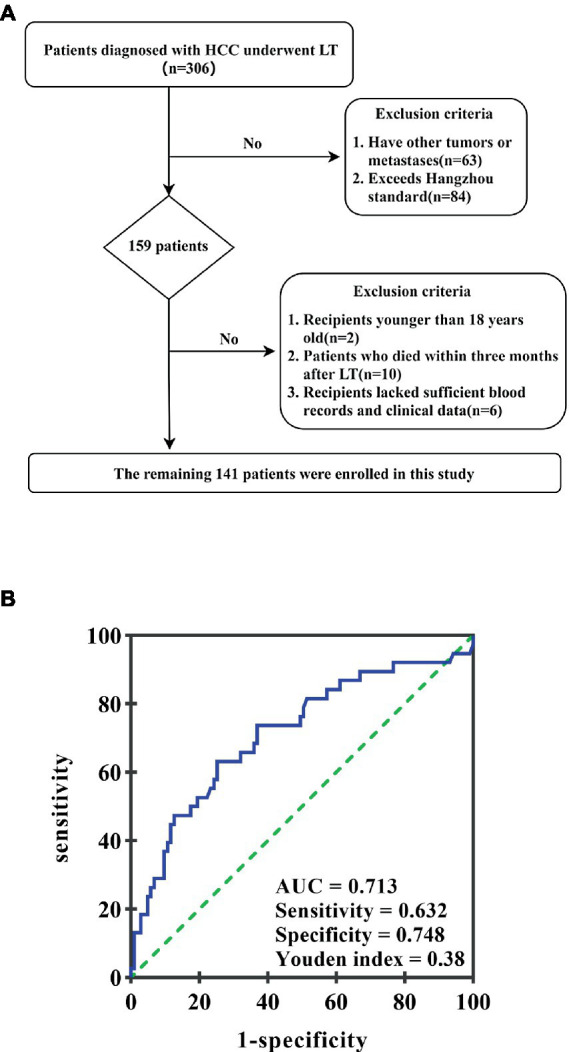
**(A)** The patient selection flowchart. **(B)** ROC curve of the GAR value in predicting HCC recurrence after liver transplantation. The area under the ROC curve is 0.713. GAR value 2.04 is considered the best cutoff value; its highest Youden index is 0.38; sensitivity and specificity are 63.2 and 74.8%, respectively.

This retrospective study included 306 consecutive patients diagnosed with HCC between June 2017 and November 2020 at the Hepatobiliary and Pancreatic Surgery Department of Shulan (Hangzhou) Hospital, Affiliated with Zhejiang Shuren University Shulan International Medical College, China. All patients underwent LT, with subsequent pathological confirmation of HCC. Inclusion criteria were: (1) pathological HCC diagnosis after the first orthotopic LT, (2) absence of other tumors or metastases, and (3) adherence to the Hangzhou criteria. Patients were excluded if they had, (1) other tumors or metastases (*n* = 63), (2) exceeded the Hangzhou criteria (*n* = 84), (3) were younger than 18 years old (*n* = 2), (4) died within three months after LT (*n* = 10), or (5) lacked sufficient blood records and clinical data (*n* = 6) (Figure 1A). Ultimately, 141 patients were enrolled in this study.

### Data collection methods

Electronic medical records were used to collect clinical information from enrolled patients. The primary data consisted of age, gender, tumor size, hepatitis virus infection, tumor number, preoperative serum albumin, differentiation, and GGT. The GGT-to-serum albumin ratio was calculated by dividing preoperative gamma-glutamyl transpeptidase by albumin. To determine the best cutoff value of preoperative GAR for predicting post-transplantation long-term survival, ROC analysis and the Youden index were used. When the area under the receiver operating characteristic (ROC) curve was 0.713, the Youden index achieved its highest value, resulting in a sensitivity of 63.2% and a specificity of 74.8%. Consequently, a GAR of 2.04 was identified as the optimal critical value (Figure 1).

### Follow-up

All transplant recipients were followed closely on an outpatient basis. The average follow-up duration was 46.6 months, ranging from 9.6 to 75.3 months. During the first 6 months after surgery, physical examinations and laboratory tests were performed monthly for each patient. This frequency decreased to every 3–6 months for the next 2 years and biannually thereafter. Contrast-enhanced abdominal computed tomography (CT) or magnetic resonance imaging (MRI) scans were performed routinely every 6 months. If local recurrence or distant metastasis was suspected, specific imaging examinations, including CT, MRI, bone scans, and positron emission tomography-computed tomography (PET-CT), were conducted promptly. Overall survival (OS) was defined as the time between LT and either death or the last follow-up. Recurrence-free survival (RFS) was defined as the time from LT to tumor recurrence or the last follow-up.

## Statistical analysis

Two clinicians independently completed the follow-up and data review. Categorical variables were presented as numbers and percentages. They were compared using either Pearson’s chi-square analysis or Fisher’s exact test, depending on which was more appropriate. For continuous variables, Student’s t-test was used for normally distributed data, while the Mann–Whitney rank sum test was used for non-normally distributed data. ROC curve analysis was used to determine the optimal GAR cutoff value for predicting post-transplantation survival and recurrence of HCC patients. The cutoff value corresponding to the highest Youden index was considered optimal. Kaplan–Meier estimation and log-rank tests were used to compare OS and disease-free survival between recipients in the high and low GAR ratio groups. Univariate and multivariate analyses were conducted using the Cox proportional hazards model to identify significant prognostic factors. All statistical tests were two-sided, with a significance level set at a *p*-value of <0.05. Data analysis was performed using SPSS software (Version 27.0, Chicago, IL, United States) and GraphPad Prism (Version 8.0, San Diego, CA, United States).

## Results

### Patient baseline clinical characteristics

The 141 eligible HCC patients were categorized into high-risk (GAR ≥2.04, *n* = 50) and low-risk (GAR <2.04, *n* = 91) groups based on the optimal cutoff value. Table 1 summarizes the baseline characteristics of all enrolled patients. Among the patients, 92.2% (*n* = 130) were male and 7.8% (*n* = 11) were female. A total of 57 patients had microvascular invasion (MVI). According to the AJCC Version 8 TNM staging system (13), 30 patients were classified into stage I + II and 111 into stage III. The Barcelona Clinic Liver Cancer (BCLC) system (14) categorized 84 patients as 0 + A and 57 as B + D. Additionally, 44.7% (*n* = 63) of patients were over the median age of 55, 34% (*n* = 48) had a BMI greater than 25, and 83.7% (*n* = 118) had a total tumor size of ≤8 cm. Approximately half (49.6%, *n* = 70) of the patients had ascites before surgery, 95.7% (*n* = 135) of the patients had liver cirrhosis, and 80.1% (*n* = 113) were positive for hepatitis B preoperatively. Significant differences in tumor-related characteristics were observed between the GAR groups, including tumor number, total tumor diameter, and compliance with the Milan, UCSF, or Hangzhou criteria (*p* < 0.05). Interestingly, GAR was not significantly associated with MELD score (>20) (*p* = 0.291), as well as histopathological characteristics such as tumor differentiation (*p* = 0.162), liver cirrhosis (*p* > 0.999), and maximum tumor diameter (>5 cm) (*p* = 0.093).

**Table 1:** Comparison of clinical characteristics between GAR ≥ 2.04 and GAR < 2.04 groups.

Variables	All patients (*n* = 141)	GAR grade		*p*-value
		Low (*n* = 91)	High (*n* = 50)	
Gender (Male/Female)	130/11	82/9	48/2	0.328
Age, years (>55/≤55)	63/78	38/53	25/25	0.346
BMI, kg/m^2^ (>25/≤25)	48/93	35/56	13/37	0.135
Smoking (Yes/No)	61/80	38/53	23/27	0.627
Alcohol (Yes/No)	54/87	34/57	20/30	0.758
MVI (Yes/No)	57/84	31/60	26/24	**0.038**
Tumor number (Multiple/Single)	78/63	43/48	35/15	**0.009**
Maximum tumor size, cm (>5/≤5)	19/122	9/82	10/40	0.093
Total tumor size, cm (>8/≤8)	23/118	9/82	14/36	**0.005**
TNM stages (III/I-II)	30/111	17/74	13/37	0.310
BCLC stages(B+D/0+A)	57/84	41/50	16/34	0.153
AFP, ng/mL (>400/≤400)	18/123	10/81	8/42	0.394
Liver cirrhosis (Yes/No)	135/6	87/4	48/2	>0.999
MELD score (>20/≤20)	20/121	15/76	5/45	0.291
Differentiation (Moderate/Well)	103/38	70/21	33/17	0.162
Positive HBsAg (+/−)	113/28	72/19	41/9	0.682
Ascites (+/−)	70/71	47/44	23/27	0.521
Milan criteria (Yes/No)	80/61	64/27	16/34	**<0.001**
UCSF criteria (Yes/No)	90/51	70/21	20/30	**<0.001**
Hangzhou criteria (A/B)	118/23	82/9	36/14	**0.005**

Bold values are *p* < 0.05.

### Prognostic significance of GAR on short- and long-term outcomes

The prognostic significance of GAR for short- and long-term outcomes was assessed. Among HCC patients meeting the Hangzhou criteria (*n* = 141), 56.7% (*n* = 80) met the Milan criteria, and 63.8% (*n* = 90) met the UCSF criteria. The median follow-up duration for all patients was 48 months (range: 9.6–75.3 months), during which 41 patients (38%) were confirmed to have died, and 47 patients (43.5%) had confirmed recurrences. The median OS stood at 48 months, accompanied by 1-, 3-, and 5-year OS rates of 95.7, 77.3, and 70.6%, respectively. Concurrently, the median RFS time was 44.58 months, with RFS rates at 1, 3, and 5 years recorded as 80.9, 72.9, and 70.9%, respectively.

**Figure 2:**
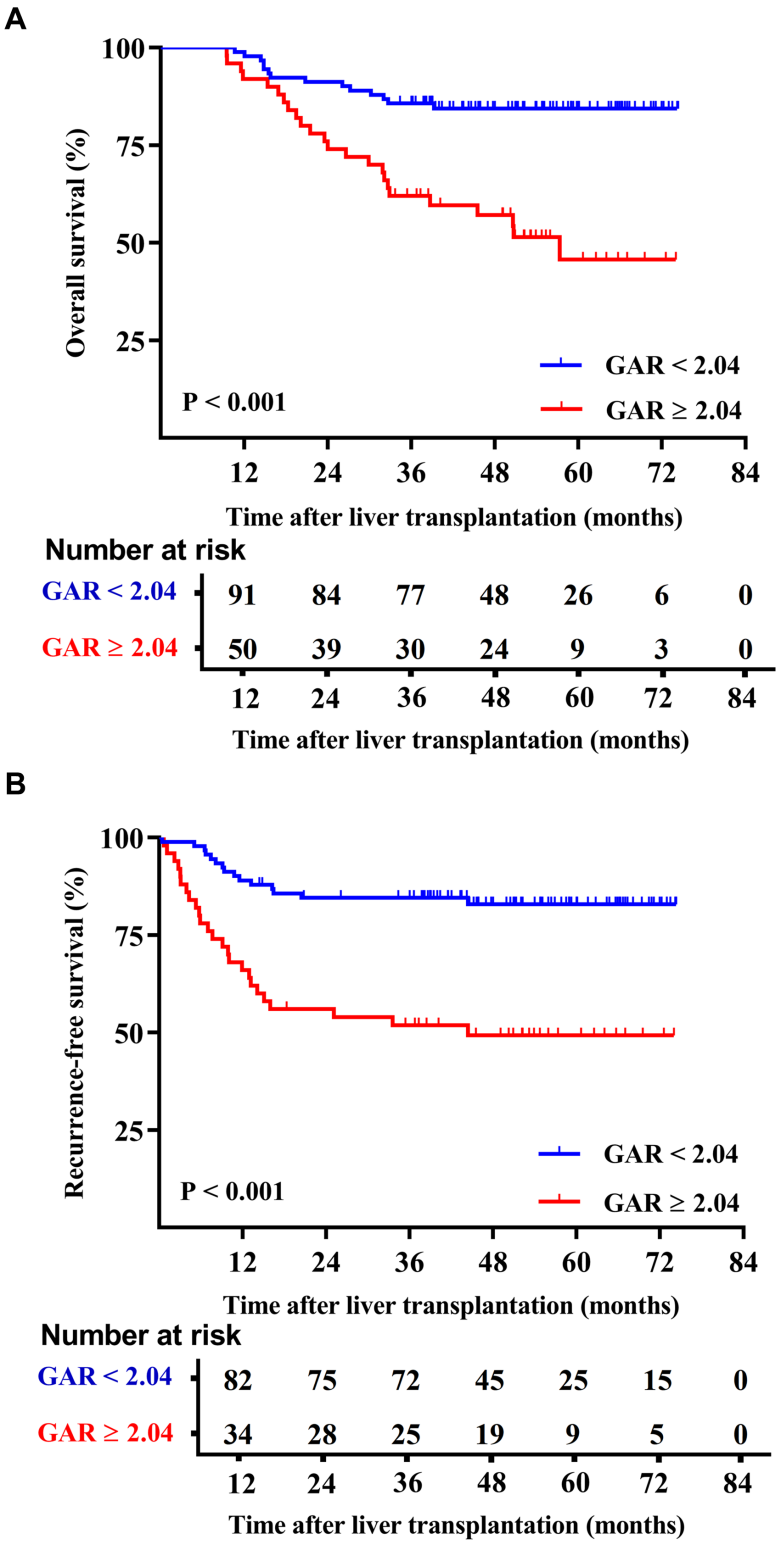
Comparison of overall patient survival and recurrence-free survival between GAR ≥ 2.04 and <2.04 patients. The patients were divided into two groups according to the pre-transplant GAR cutoff value of 2.04. Recipients in the GAR < 2.04 group presented significantly higher OS rates [*p* < 0.001 **(A)**] and RFS rates [*p <* 0.001 **(B)**] than those in the GAR ≥ 2.04 group.

The Kaplan–Meier survival curves indicated that the low GAR group exhibited significantly higher 1-, 3-, and 5-year OS rates in contrast to the high GAR group (98.9, 85.7, and 84.4% vs. 92.0, 62.0, and 51.4%, respectively; *p* < 0.001, Figure 2A). Similarly, at 1-, 3-, and 5-year post-surgery, the low GAR group demonstrated significantly elevated RFS rates compared to the high GAR group (89.0, 84.5, and 82.9% vs. 66.0, 51.9, and 49.3%, respectively; *p* < 0.001, Figure 2B).

**Figure 3:**
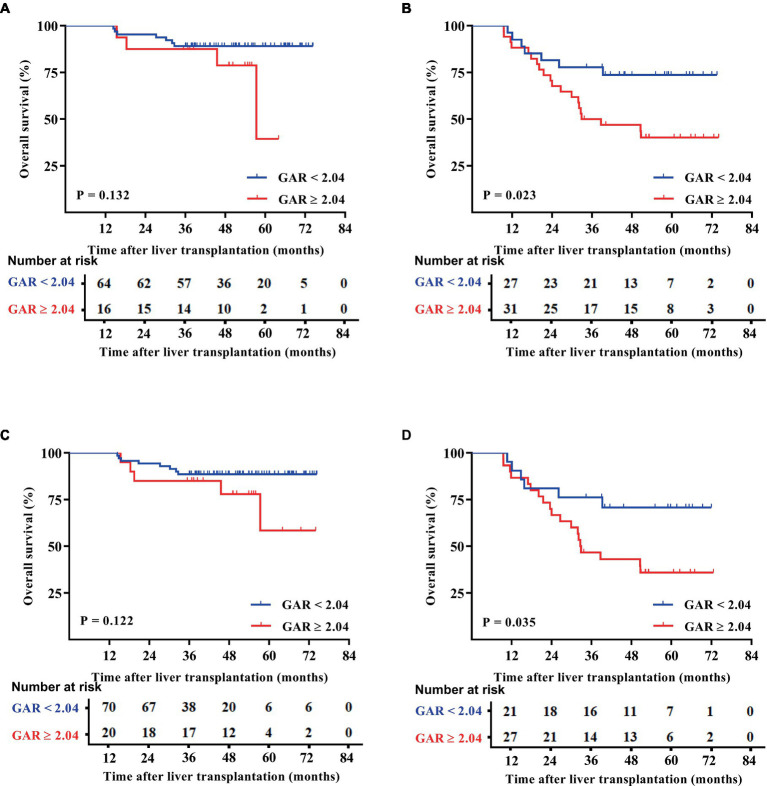
OS outcomes of patients were divided according to the Milan and UCSF criteria. The patients were divided into the Milan **(A)** and UCSF **(C)** criteria, beyond Milan **(B)** and UCSF **(D)** criteria. The OS of the Milan,  UCSF, and GAR < 2.04 groups was comparable with that of the GAR ≥ 2.04 group, and no statistically significant difference was presented [*p >* 0.05 **(A,C)**]. Patients in the beyond Milan and UCSF criteria and GAR < 2.04 groups had significantly higher OS than the GAR ≥ 2.04 group [*p <* 0.05 **(B,D)**].

**Figure 4:**
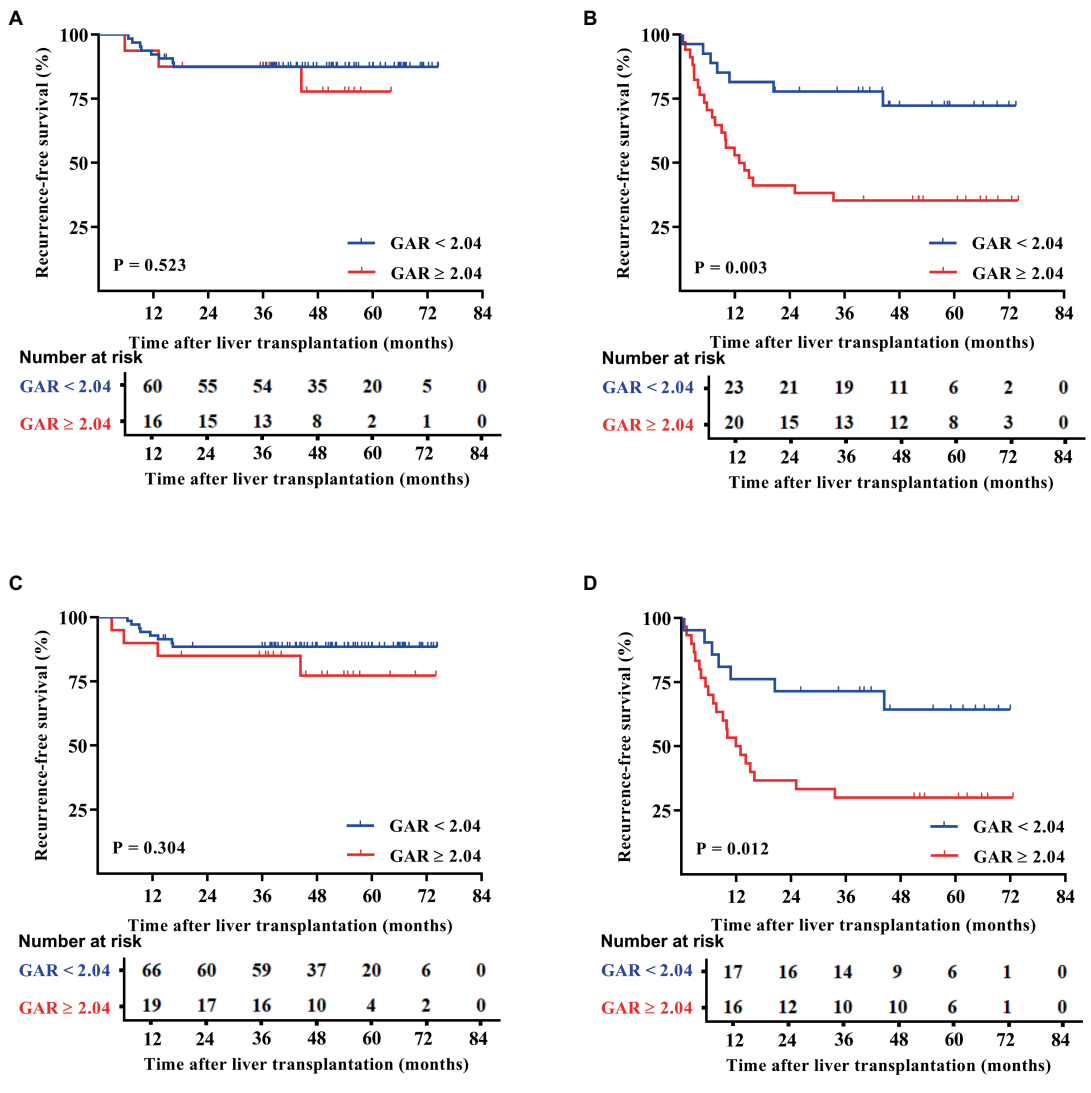
RFS outcomes of patients were divided according to the Milan and UCSF criteria. The patients were divided into the Milan **(A)** and UCSF **(C)** criteria, beyond Milan **(B)**, and UCSF **(D)** criteria. The RFS of the Milan or UCSF and GAR < 2.04 groups was comparable with that of the GAR≥2.04 group, and no statistically significant difference was presented [*p >* 0.05 **(A,C)**]. Patients in the beyond Milan and UCSF criteria and GAR < 2.04 groups had significantly higher RFS than the GAR ≥ 2.04 group [*p <* 0.05 **(B,D)**].

Patients were categorized based on whether they met or exceeded the Milan and UCSF criteria. Interestingly, the results showed that within both criteria groups, patients with a GAR of 2.04 or higher (high GAR) had a significantly poorer prognosis (Figures 3, 4). However, when looking specifically at patients who met the Milan or UCSF criteria (within criteria), there were no significant differences in OS or RFS between the high GAR and low GAR groups (*p* > 0.05; Figures 3A,C, 4A,C). In contrast, for patients who exceeded the Milan or UCSF criteria (beyond criteria, specifically standard or UCSF standard group), a high GAR was significantly associated with worse OS and RFS, highlighting its impact on the prognosis of HCC patients after transplantation (*p* < 0.05; Figures 3B,D, 4B,D).

**Figure 5:**
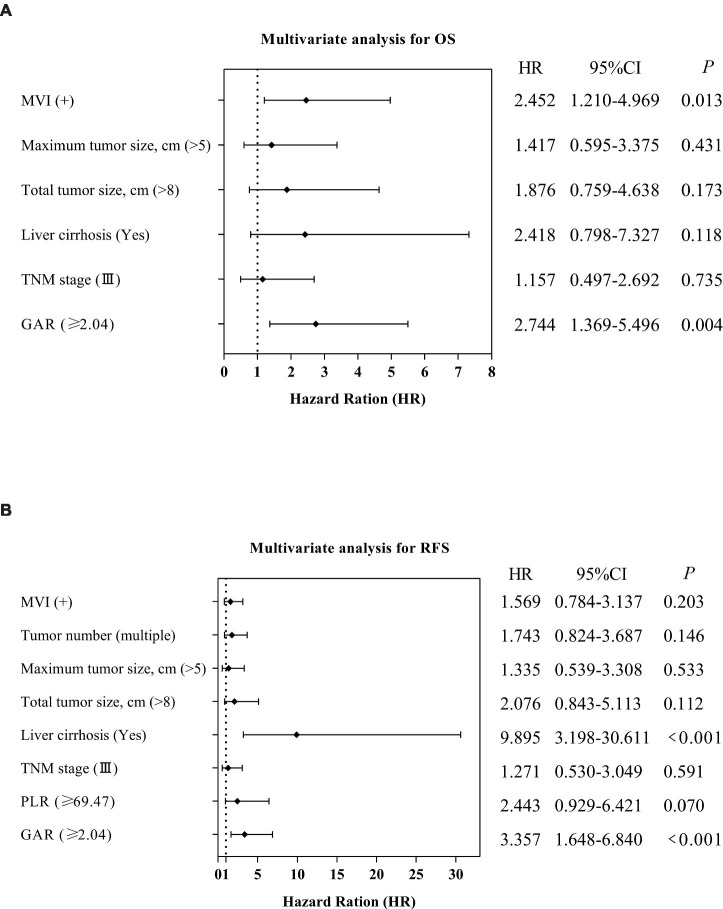
Forest plot comparing independent risk factors affecting OS and RFS after transplantation in HCC patients according to multivariate analysis.

Following the outcomes of univariate analysis, maximum tumor size (>5 cm) [hazard ratio (HR): 3.143; 95% confidence interval (CI): 1.556–6.349; *p* = 0.001], total tumor size (>8 cm) (HR: 3.580; 95% CI: 1.824–7.028; *p* < 0.001), MVI (Yes) (HR: 3.349; 95% CI: 1.712–6.551; *p* < 0.001), TNM stage (III) (HR: 2.522; 95% CI: 1.303–4.802; *p* = 0.006), and GAR (≥2.04) (HR: 3.685; 95% CI: 1.903–7.133; *p* < 0.001) demonstrated significant associations with OS (Table 2). To minimize the potential interactions among these variables, significant variables in the univariate Cox regression analysis were identified and incorporated into the multivariate Cox proportional hazards model. Following the multivariate analysis, MVI(Yes) (HR: 2.452; 95% CI: 1.210–4.969; *p* = 0.013), and pre-LT serological test ratios, only GAR appeared as an independent risk factor for OS (HR: 2.744; 95% CI: 1.369–5.496; *p* = 0.004) (Table 2; Figure 5A).

**Table 2:** Univariate and multivariate cox analyses show prognostic factors for OS.

Variables	Univariate analysis			Multivariate analysis		
	HR	(95% CI)	*p*-value	HR	(95% CI)	*p*-value
Gender (Male/Female)	1.713	0.412–7.123	0.459			
Age, years (>55/≤55)	1.169	0.614–2.227	0.634			
BMI, kg/m^2^ (>25/≤25)	1.534	0.745–3.159	0.246			
Smoking (Yes/No)	1.052	0.553–2.004	0.877			
Alcohol (Yes/No)	1.253	0.658–2.385	0.493			
MVI (Yes/No)	3.349	1.712–6.551	**<0.001**	2.452	1.210–4.969	**0.013**
Tumor number (Multiple/Single)	1.447	0.748–2.800	0.272			
Maximum tumor size, cm (>5/≤5)	3.143	1.556–6.349	**0.001**	1.417	0.595–3.375	0.431
Total tumor size, cm (>8/≤8)	3.580	1.824–7.028	**<0.001**	1.876	0.759–4.638	0.173
AFP, ng/mL (>400/≤400)	1.308	0.546–3.130	0.547			
Liver cirrhosis (Yes/No)	3.581	1.263–10.152	**0.016**	2.418	0.798–7.327	0.118
MELD score (>20/≤20)	1.156	0.451–2.963	0.763			
Differentiation (Moderate/Well)	1.441	0.660–3.146	0.359			
TNM stage (III/I+II)	2.522	1.303–4.802	**0.006**	1.157	0.497–2.692	0.735
BCLC stage (B+D/0+A)	1.517	0.775–2.968	0.223			
Positive HBsAg (+/−)	1.404	0.587–3.359	0.446			
Ascites (+/−)	1.197	0.631–2.269	0.582			
NLR grade (≥2.59/<2.59)	1.334	0.682–2.607	0.400			
PLR grade (≥69.47/<69.47)	2.187	0.963–4.969	0.062			
AAPR grade (≥0.58/<0.58)	1.057	0.442–2.528	0.902			
ALBI grade [≥(−2.26)/<(−2.26)]	1.135	0.599–2.152	0.698			
GLR grade (≥86.97/<86.97)	1.355	0.711–2.580	0.356			
ALR grade (≥145.01/<145.01)	1.785	0.885–3.599	0.105			
GAR grade (≥2.04/<2.04)	3.685	1.903–7.133	**<0.001**	2.744	1.369–5.496	**0.004**

Bold values are *p* < 0.05.

Similarly, we performed a multivariate Cox analysis aimed at identifying prognostic factors for RFS (Table 3). We found that liver cirrhosis (Yes) (HR = 9.895; 95% CI: 3.198–30.611; *p* < 0.001) and GAR (HR = 3.357; 95% CI: 1.648–6.840; *p* < 0.001) were associated with a higher risk of RFS (Table 3; Figure 5B).

**Table 3:** Univariate and multivariate cox analyses show prognostic factors for RFS.

Variables	Univariate analysis			Multivariate analysis		
value	HR	(95% CI)	*p*-value	HR	(95% CI)	*p*-value
Gender (Male/Female)	1.679	0.405–6.960	0.440			
Age, years (>55/≤55)	1.150	0.614–2.153	0.663			
BMI, kg/m^2^ (>25/≤25)	1.890	0.900–3.971	0.093			
Smoking (Yes/No)	1.149	0.610–2.164	0.667			
Alcohol (Yes/No)	1.410	0.756–2.630	0.280			
MVI (Yes/No)	2.993	1.577–5.680	**<0.001**	1.569	0.784–3.137	0.203
Tumor number (Multiple/Single)	2.471	1.234–4.948	**0.011**	1.743	0.824–3.687	0.146
Maximum tumor size, cm (>5/≤5)	2.836	1.384–5.812	**0.004**	1.335	0.539–3.308	0.533
Total tumor size, cm (>8/≤8)	4.494	2.359–8.561	**<0.001**	2.076	0.843–5.113	0.112
AFP, ng/mL (>400/≤400)	1.312	0.551–3.126	0.540			
Liver cirrhosis (Yes/No)	7.473	2.887–19.343	**<0.001**	9.895	3.198–30.611	**<0.001**
MELD score (>20/≤20)	1.005	0.422–2.395	0.990			
Differentiation (Moderate/Well)	1.297	0.617–2.725	0.492			
TNM stage (III/I+II)	2.801	1.475–5.318	**0.002**	1.271	0.530–3.049	0.591
BCLC stage (B+D/0+A)	1.572	0.811–3.049	0.181			
Positive HBsAg (+/−)	1.501	0.630–3.574	0.359			
Ascites (+/−)	1.121	0.602–2.085	0.719			
NLR grade (≥2.59/<2.59)	1.567	0.808–3.037	0.184			
PLR grade (≥69.47/<69.47)	3.912	1.532–9.989	**0.004**	2.443	0.929–6.421	0.070
AAPR grade (≥0.58/<0.58)	1.831	0.891–3.748	0.098			
AIBI grade [≥(−2.26)/< (−2.26)]	1.413	0.760–2.628	0.275			
GLR grade (≥86.97/<86.97)	1.043	0.545–1.998	0.899			
ALR grade (≥145.01/<145.01)	1.694	0.848–3.391	0.137			
GAR grade (≥2.04/<2.04)	3.859	2.032–7.328	**<0.001**	3.357	1.648–6.840	**<0.001**

Bold values are *p* < 0.05.

Our comprehensive analysis results demonstrate that high GAR values in preoperative non-invasive serum tests (HR: 2.744, *p* = 0.004 for OS; HR: 3.357, *p* < 0.001 for RFS) function as independent prognostic factors for adverse OS and RFS, as illustrated in Figure 5.

**Figure 6:**
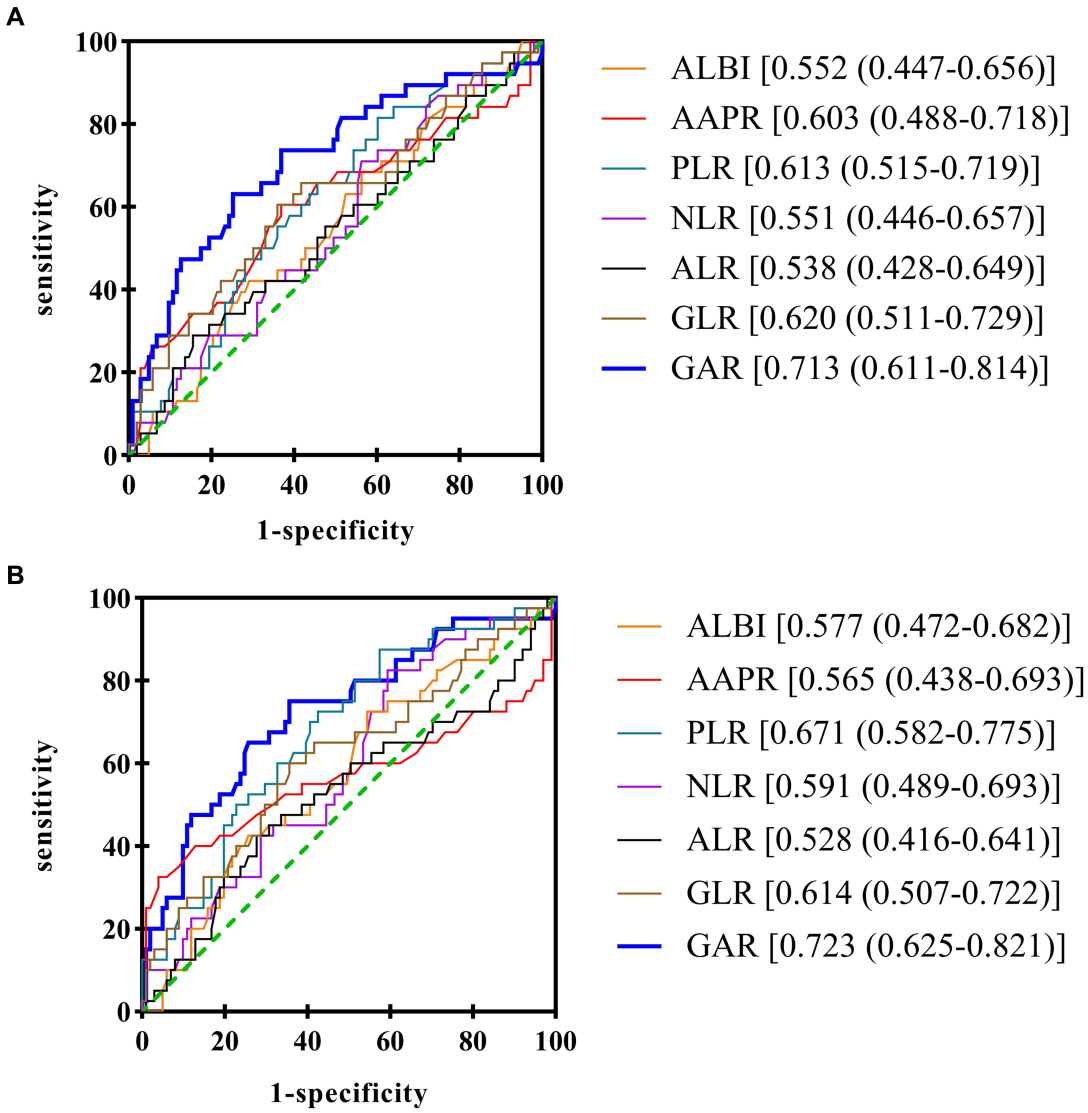
Comparison of the predictive accuracy of GAR and other variables in the derivation cohort **(A)** OS, overall survival **(B)** RFS, recurrence-free survival.

We conducted a deeper investigation into the relationship between the serum albumin/alkaline phosphatase ratio (AAPR), the albumin–bilirubin (ALBI) grading system, the γ-glutamyl transferase to lymphocyte count ratio (GLR), and the aminotransferase-to-lymphocyte ratio (ALR). These parameters have previously been examined in patients who have undergone liver transplantation or those who have undergone resection for HCC (15–18), NLR, PLR, and ALBI with clinicopathological features and their ability to predict survival outcomes using the same methods described above (10). ROC curve analysis was used to compare the accuracy of these markers in predicting the prognosis of patients with HCC who met the Hangzhou criteria for LT. The Youden index calculations determined the optimal cutoff points: −2.26 for ALBI grade, 0.58 for AAPR, 69.47 for PLR, 86.97 for GLR, 145.01 for ALR, and 2.59 for NLR. The AUCs of OS for ALBI, AAPR, PLR, NLR, ALR, and GLR were 0.552, 0.603, 0.613, 0.551, 0.538, and 0.620, respectively (Figure 6A). The AUCs of RFS for ALBI, AAPR, PLR, NLR, ALR, and GLR were 0.577, 0.565, 0.671, 0.591, 0.528, and 0.614, respectively (Figure 6B).

## Discussion

HCC incidence and mortality rates vary significantly worldwide due to factors such as genetics, environment, lifestyle, and infections. LT remains a crucial treatment for HCC patients (1). Predicting the prognosis for patients with HCC is essential. While a biopsy is the standard for diagnosing HCC and MVI, it also carries risks such as bleeding and needle tract tumor spread. In contrast, serum tumor markers such as AFP offer a non-invasive and reproducible approach for early diagnosis (1). Our data demonstrated that the GAR score, which involves serum GGT and serum protein levels, is a simple and convenient ratio with significant predictive value. Previous studies have shown the importance of these serum markers in tumor progression (14, 15).

GGT plays a crucial role in glutathione metabolism. It not only protects cells from oxidative damage but also contributes to oxidative stress, impacting proliferation, apoptosis, and immune responses. The activity of GGT primarily reflects the extent of harm to liver and bile duct cells (19, 20), which is associated not only with HCC but also with the digestive system, respiratory tract, breast, and lymphoma, affecting the risk of cancer in these systems (21–23). In the context of HCC treatment, such as liver resection and LT, an increase in GGT is considered an indicator of a poor prognosis (24–26). Similarly, serum albumin, reflecting liver health and nutritional status, serves as a potent free radical scavenger, antioxidant, and immune regulator, in addition to its role in transporting substances and maintaining blood vessel pressure. It interacts with various substances, including metal ions, toxic metabolites, and inflammatory mediators, thereby influencing the body’s inflammatory and antioxidant responses (27–29), and various pathological conditions such as malnutrition, weakened immune defenses, and reduced cell function are closely interconnected with these factors (29, 30). Furthermore, the latest Meld 3.0 version incorporates serum albumin into its calculations to enhance mortality prediction accuracy (31).

Current evidence suggests that GGT and serum albumin play a vital role in assessing liver function, nutritional status, and inflammatory response. Their value in predicting prognoses, particularly for cancer, is undeniable. Tumor-associated inflammation, often induced by innate immune cells, can promote tumor growth while suppressing adaptive immune responses—a key area of cancer research and drug development. Oncogenic mutations and signaling alterations in cancer cells can further exacerbate this inflammation by affecting chemokines, cytokines, tissue structure, oxygen pressure, and microbial translocation (32). In the context of basic research, some researchers have combined these two indicators and demonstrated that the GAR holds significant predictive value in assessing the prognosis of numerous hepatobiliary disease-related disorders. For instance, GAR can predict liver fibrosis and cirrhosis in patients with chronic hepatitis B (33), and it is strongly associated with the prognosis of liver resection in cirrhosis and HCC cases (12, 34). Post-surgery, high GAR values often correlate with reduced OS and RFS (10, 35). Moreover, GAR has shown predictive value for independent prognosis in patients undergoing pancreatic ductal adenocarcinoma surgery (11). Nonetheless, its predictive potential in LT surgery research warrants further exploration, given that it has not been extensively investigated. When combined with our findings from this research, it was found that GAR could identify high-risk HCC transplant patients. Prior to LT for cirrhosis or HCC, effective serological screening and selection can reduce the risk of post-transplant HCC recurrence, thereby improving the quality of life for patients and increasing their survival rates. These findings further underscore the importance of GAR in pre-transplant evaluation. However, the specific mechanism is unclear and requires further investigation.

Upon retrospective data analysis, this study revealed that patients with low GAR ratios following HCC transplantation based on Hangzhou standards exhibited better outcomes in terms of survival and tumor-free survival compared to those with high GAR ratios. High GAR values may suggest a weakened tumor resistance mechanism, potentially leading to poorer treatment outcomes for patients. While the reasons behind the increased risk of tumor recurrence and death following LT with higher GAR values remain unclear, they may shed light on previously established biological functions. Despite the numerous scoring systems and prognostic models suggested previously, such as NLR (8), PLR (9), AAPR (15), ALBI, and the systemic inflammatory response index (SIRI) (16, 36). GLR and ALR have been studied in liver transplant patients or HCC after resection patients (17, 18). These scoring systems or models hold the potential for assessing pre-surgery liver function and inflammation in LT patients. Nevertheless, their clinical applicability and accuracy currently lack standardization and remain to be validated and improved in terms of prognostic evaluation.

Our study has several limitations. First, it is a retrospective, single-center study conducted domestically with a limited sample size. Second, there may be variations in surgical techniques and perioperative management among transplant patients. Third, the study evaluated the prognostic value of GAR only in HCC patients who underwent LT and did not assess its potential role in patients receiving downstage therapy. Finally, the lack of external validation for our findings raises the possibility of selection bias. To address these limitations and strengthen our conclusions, future investigations should be large-scale, multicenter, prospective studies with diverse patient populations.

## Conclusion

This study investigated the potential of the pre-LT GGT-to-serum albumin ratio as a prognostic marker for HCC patients undergoing LT who meet the Hangzhou criteria. Our findings suggest that a pre-transplant GAR of 2.04 or higher is an independent predictor of prognosis and survival outcomes after LT for HCC. GAR is a simple and cost-effective laboratory test with the advantages of being non-invasive and reproducible. For patients who satisfy the Hangzhou criteria, the preoperative GAR offers additional prognostic data relevant to liver transplant outcomes. Moreover, a GAR value lower than 2.04 may be indicative of increased suitability for LT.

## Data availability statement

The raw data supporting the conclusions of this article will be made available by the authors, without undue reservation.

## Ethics statement

This study was approved by the Human Research Ethics Committee of Shulan (Hangzhou) Hospital (No: KY2024020), and complies with the principles of the Declaration of Helsinki of the World Medical Association.

## Author contributions

X-YL: Writing – original draft, Writing – review & editing, Conceptualization, Data curation, Formal analysis, Investigation, Methodology, Validation. K-WC: Writing – original draft, Writing – review & editing, Conceptualization, Data curation, Formal analysis, Investigation, Validation. NY: Data curation, Methodology, Writing – original draft, Writing – review & editing. C-HG: Data curation, Methodology, Writing – original draft, Writing – review & editing. Q-BZ: Conceptualization, Data curation, Formal analysis, Investigation, Writing – original draft, Writing – review & editing. J-PL: Conceptualization, Data curation, Software, Supervision, Writing – original draft, Writing – review & editing. XZ: Formal analysis, Investigation, Supervision, Validation, Writing – original draft. S-SZ: Funding acquisition, Project administration, Resources, Software, Supervision, Validation, Visualization, Writing – review & editing. ZY: Funding acquisition, Methodology, Project administration, Resources, Supervision, Validation, Visualization, Writing – review & editing.
